# Kinetics overcome thermodynamics in primitive analogs of the reverse tricarboxylic acid cycle

**DOI:** 10.1039/d5sc05872d

**Published:** 2026-02-04

**Authors:** Vignesh Sathyaseelan, John Morgan, Brett M. Savoie

**Affiliations:** a Davidson School of Chemical Engineering, Purdue University West Lafayette Indiana 47906 USA jamorgan@purdue.edu bsavoie2@nd.edu; b Department of Chemical and Biomolecular Engineering, University of Notre Dame Notre Dame Indiana 46556 USA

## Abstract

The reverse/reductive tricarboxylic acid (rTCA) cycle is a metabolic pathway that facilitates CO_2_ fixation in certain anaerobic bacteria and archaea. Its presence in phylogenetically ancient organisms has led to hypotheses about its role in the early evolution of CO_2_ fixation pathways. While the thermodynamics of the pathway is well studied, the kinetic feasibility of uncatalyzed rTCA cycle reactions remains uncertain. In this study, we report a systematic, mechanistic, and kinetic characterization of the uncatalyzed rTCA cycle and its side reactions. A primitive, uncatalyzed rTCA reaction network is elucidated for carbon dioxide fixation that includes all transition states and water-catalyzed reaction channels. The thermodynamics and kinetics of competing off-cycle reactions and potential cycles that are parallel to the uncatalyzed rTCA cycle are also investigated using a newly developed chemical reaction network exploration method. While previous work examined overall thermodynamics and possible pathways, our work focuses on kinetic bottlenecks, which guide where in nature to search for primordial catalysts (clays, minerals, *etc.*) that could lower the major transition state barriers. This exploration reveals that the uncatalyzed rTCA pathway lies in a reaction neighborhood that is thermodynamically favored, but several key steps are kinetically challenging in the absence of catalysis owing to competitive intramolecular side-reactions and the absence of favorable parallel cycles. The kinetic modeling also provides intermediates that would accumulate in an uncatalyzed environment, serving as prebiotic signposts.

## Abstract 2

The reverse/reductive tricarboxylic acid (rTCA) cycle is a highly conserved CO_2_ fixation pathway and candidate for prebiotic carbon fixation. The kinetic viability of prebiotic hypotheses are studied here using reaction exploration algorithms.

## Graphical abstract

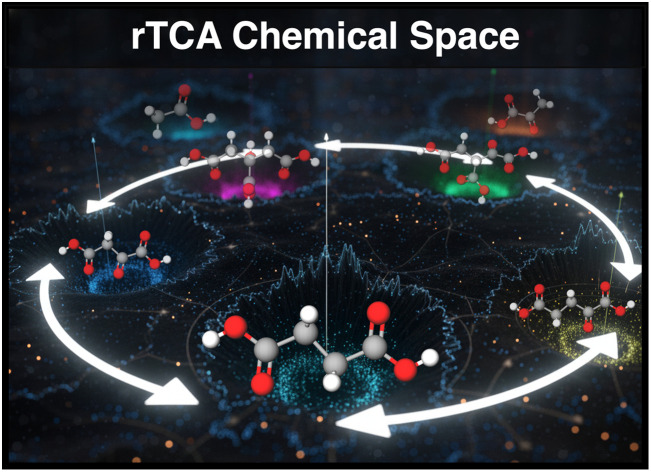

## 1. Introduction

The reverse tricarboxylic acid (rTCA) cycle, also known as the reductive citric acid cycle, is an important metabolic pathway found in several anaerobic bacteria and archaea to fix carbon dioxide.^1^ This cycle has been proposed as a possible prebiotic pathway for uncatalyzed carbon fixation, owing to both its simplicity and its presence in phylogenetically ancient organisms.^2–5^ However, evolutionary history makes it challenging to infer prebiotic chemical pathways from modern biochemical processes, as these pathways may have been substantially changed. Consequently, prebiotic chemistry may have been fundamentally distinct from the biochemistry observed in extant organisms.^6^ While the rTCA cycle offers a plausible route for early autotrophic metabolism, its prebiotic role remains unclear. Previous studies have established its thermodynamic favorability, but understanding the kinetics of its uncatalyzed reactions is crucial for evaluating whether this pathway could have operated prior to the evolution of enzymes, particularly in the presence of competing side reactions.^7^ The short-lived nature of intermediates and the large number of potential off-target reactions make experimental mechanistic studies challenging, but recently developed computational approaches make it timely to revisit these gaps.^7–10^

Early computational studies of the rTCA cycle focused on whether it was unique or representative of a larger family of possible cycles. Morowitz *et al.* found that while the 11 molecules comprising the rTCA cycle are among a subset of organic chemical space that satisfies a set of feasibility criteria for primitive carbon fixation, they are not unique in doing so.^9^ Over a hundred other known molecules also satisfy these constraints, comprising about 0.004% of the relevant organic chemical space. Other researchers later extended this approach by enumerating additional chemical subspaces in search of rTCA alternatives.^8,11^ These findings suggest that multiple alternative carbon fixation cycles might have been chemically possible in a prebiotic context. However, these simple filters do not capture the full requirements of a plausible prebiotic carbon fixation cycle. Alternative pathways may have faced insurmountable kinetic or thermodynamic barriers under early Earth conditions or may have been less accessible to subsequent evolutionary optimization. Understanding these constraints is crucial for determining why the rTCA cycle, among many theoretically possible options, became a central feature of modern metabolism.

The first step in this direction was taken by Zubarev *et al.*, who constructed a superset of carbon fixation cycles using a small set of reaction rules applied to acetate, H_2_O, CO_2_, and H_2_.^7^ This exploration rediscovered the rTCA cycle, but more surprisingly, it also revealed a large set of parallel and partially overlapping fixation cycles (*i.e.*, sharing some reactions with the rTCA cycle but many independent) that are also thermodynamically favorable. This clarified that many thermodynamically favorable CO_2_ fixation cycles could exist, provided that H_2_ is available as a reductant, but still left unaddressed the central question of whether any of these alternatives are kinetically relevant.

The rTCA cycle's widespread conservation across life suggests it represents a crucial evolutionary innovation, yet these computational analyses show it as just one of many possible solutions. The apparent paradox may be resolved by considering previously overlooked kinetic factors. These reactions may be thermodynamically feasible but kinetically constrained, either through extremely high activation energies or through complex multi-step reaction mechanisms. Although many fixation cycles are theoretically possible, their practical viability depends on whether the desired reactions can proceed faster than competing side reactions. A crucial factor is also where metabolites accumulate in these cycles, as these accumulation points create vulnerability to unwanted side reactions.

Here, we systematically characterize the reaction mechanisms and kinetics of the uncatalyzed rTCA cycle and structurally related reaction networks that share key chemical transformations. The recent development of high-throughput methods for characterizing reaction kinetics now makes it possible to comprehensively investigate reaction networks at a scale that is comparable to the foregoing studies that were limited only to thermodynamic characterizations.^12–14^ In this work, the kinetics of uncatalyzed rTCA cycles and side reactions are characterized using a computational reaction exploration methodology. In this study, side reactions are defined as those that (i) originate from intermediates within the main cycle but give rise to products outside the primary reaction pathway, (ii) constitute mechanistic variants of a given transformation such as concerted *versus* stepwise pathways or (iii) involve alternative sequences of the same elementary steps that produce the same intermediates. Kinetic modeling of the resulting network confirmed the feasibility of the primitive rTCA cycle under idealized conditions but also showed that negligible carbon fixation flux results when the kinetics of competing off-cycle reactions were accounted for. The major off-cycle fluxes occur primarily at reaction steps that in modern organisms are catalyzed by enzymes. This correlation suggests two possibilities regarding prebiotic carbon fixation. The challenging kinetics at these steps might indicate that an uncatalyzed rTCA cycle was not a viable prebiotic pathway. Alternatively, these kinetically challenging steps might represent the precise locations where early catalysis became essential for enabling a primitive rTCA cycle. These mechanistic analyses identify specific kinetic barriers in the rTCA cycle that would require catalysts for sustained carbon fixation. Such barriers persist despite the previously reported thermodynamic favorability of numerous rTCA analogs, indicating that thermodynamic analysis alone provides insufficient evidence for evaluating prebiotic reaction networks.

## 2. Results & discussions

The Yet Another Reaction Program (YARP) provided a systematic approach for characterizing transition states across the complete primitive uncatalyzed rTCA cycle network, including both main cycle reactions and competing side reactions. This automated exploration was necessary given the large number of possible reaction pathways. YARP has been used to discover organic reaction sequences in many chemical contexts.^12,15,16^ YARP uses general graphical rules to generate potential products and double-ended transition state searches to localize and compare transition states of competing reactions. YARP uses generic graph-based reaction rules involving a fixed number of bond breaks and bond formation steps to explore potential side reactions. Transition state (TS) localization calculations were performed using two complementary approaches to model the aqueous environment. The first incorporated both an explicit water molecule near reactive centers and a PCM solvation model. The second used only the PCM model, which approximates solvent effects through a continuous dielectric medium. This dual approach allows the evaluation of specific water-mediated mechanisms while maintaining a complete description of bulk solvation effects.^17^ The results involving explicit water molecules around the reaction are referred to as “water-catalyzed” throughout. The water-catalyzed reaction mechanism enables the transfer of one or several protons to or from water molecules in the TS and has a significant impact on the reaction energetics.^16^ From our analysis, the explicit consideration of water is crucial to resolving the low-barrier reaction pathway (see SI Section 1; the presence of water decreases the barriers by an average of 9.5 kcal mol^−1^, and 77% of the investigated reactions exhibit a barrier-lowering when an explicit water molecule is included). Analysis by mechanistic class revealed that cleavage (−13.7 kcal mol^−1^), hydrolysis (−12.8 kcal mol^−1^), dehydration (−12.4 kcal mol^−1^), and hydrogenation (−9.0 kcal mol^−1^) reactions showed the largest catalytic enhancement, consistent with water's ability to mediate proton transfer in transition states. Cyclization (−7.0 kcal mol^−1^) and carboxylation (−4.8 kcal mol^−1^) reactions were less affected. This widespread catalytic effect suggests water played a fundamental role in enabling prebiotic rTCA cycle chemistry. The presented free energies of activation represent the lowest values obtained from calculations that included and excluded explicit water molecules. The results are organized around the mechanisms of the uncatalyzed TCA cycle (Section 2.1), a comparison of these mechanisms with the major off-target pathways that were discovered, and the existence of parallel fixation cycles (Section 2.2), and kinetic analysis of the resulting network of reaction pathways (Section 2.3).

### 2.1 Mechanism of the uncatalyzed rTCA cycle

**Fig. 1:**
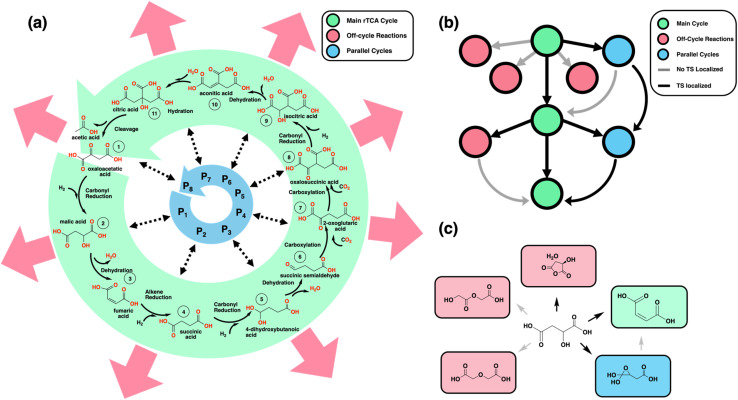
Overview of the methodology and nomenclature used for generating the rTCA reaction network. (a) Illustration of the key steps and species involved in the uncatalyzed rTCA cycle, with green arrows indicating the forward direction of carbon fixation, red arrows indicating kinetically competitive pathways that do not support fixation, and blue arrows indicating possible cycles running parallel to the uncatalyzed rTCA cycle. (b) Each circle represents a molecular species present in and around the rTCA chemical space. Black lines connect reactant–product pairs with localized transition states (TS), while grey lines denote the absence of a localized TS. Green circles correspond to species that are part of the rTCA cycle. Red circles represent outward flux from the cycle. Blue circles connected by curved arrows depict molecular species that reconnect outward flux back into the cycle or form new parallel cycles in chemical space. (c) The chemical structures of a subset of the break 2 form 2 enumeration of a molecular species in the main rTCA cycle are shown to demonstrate main cycle, off-target, and potential parallel cycle species. All off-target products are considered as potential parallel cycle species and reactions are tried to be converged, but only one product is shaded in blue to help the reader distinguish between a parallel cycle species and an off-target species.

The kinetic investigation of the uncatalyzed rTCA cycle required decomposition into mechanistically tractable elementary steps. The break-two-form-two bonds (b2f2) rule was selected because it represents the simplest mechanistic pathway capable of describing all transformations in the cycle when complex reagents and co-enzymes are replaced by prebiotic proxies such as H_2_, H_2_O, and CO_2_. A comprehensive description of the enzymatic rTCA cycle is provided by Steffens *et al.*, which was the starting point for developing our uncatalyzed enzymatic cycle.^2^ The resulting uncatalyzed cycle (Fig. 1a) is similar to that studied by Zubarev *et al.* but provides additional mechanistic details necessary for transition state analysis.^7^

The rTCA cycle can be started from any point along the cycle, but as per the numbering conventions used in this study, the cycle commences with the reduction of oxaloacetic acid^1^ to form malic acid^2^ through carbonyl reduction. This enzymatic step is facilitated by nicotinamide adenine dinucleotide (NADH) and is substituted here by H_2_. The rationale for using H_2_ is that it is a plausible prebiotic reductant with sufficient reducing power to drive the cycle under ambient conditions, consistent with previous studies.^7^ The resulting malic acid^2^ undergoes dehydration to yield fumaric acid,^3^ which then undergoes alkene reduction with the addition of H_2_ to generate succinic acid. The conversion of succinic acid^4^ to 2-oxoglutaric acid^7^ represents a key transformation that differs significantly between enzymatic and uncatalyzed pathways. While the modern enzymatic pathway employs coenzyme A (CoA) and adenosine triphosphate (ATP), the primitive uncatalyzed pathway must proceed through simpler intermediates. By substituting the CoA group with a hydrogen atom (–H), the reaction can proceed through three elementary b2f2 steps. First, H_2_ addition reduces succinate^4^ to 4-dihydroxybutanoic acid,^5^ followed by dehydration to form succinic semialdehyde,^6^ and finally, CO_2_ addition generates 2-oxoglutaric acid.^7^ This decomposition illustrates the substitutions necessary to still effect the non-catalyzed cycle in a prebiotic environment. 2-Oxoglutaric acid^7^ then undergoes a subsequent two-step reaction of carboxylation by CO_2_ addition to form oxalosuccinic acid^8^ and carbonyl reduction by H_2_ addition to form isocitric acid^9^ in the uncatalyzed cycle. In the enzymatic counterpart, this reaction uses nicotinamide adenine dinucleotide phosphate (NADPH) and a proton. In the uncatalyzed cycle, the isocitric acid^9^ intermediate is converted to citric acid^11^ through an isomerization reaction, which is decomposed into a two-step elementary b2f2 mechanism involving dehydration to form aconitic acid^10^ followed by a hydration step to form citric acid.^11^ The final step in the cycle involves C–C bond cleavage, resulting in the formation of acetic acid and oxaloacetic acid,^1^ thereby completing the rTCA cycle.

### 2.2 Comparative analysis of primary mechanisms and off-target pathways

Each reaction along the rTCA cycle is potentially in competition with off-target reactions that can be off-cycle or contribute to parallel rTCA cycles (Fig. 1b). In addition to the reactions that belong to the rTCA cycle referred to as “main cycle” reactions throughout (green circles in Fig. 1b) we investigated the kinetic feasibility of all competing b2f2 reactions involving each reactant along the primary rTCA cycle referred to as “off-cycle” reactions throughout (red circles in Fig. 1b). The exploration of side reactions allowed the uniqueness and kinetic relevance of the primitive cycle to be evaluated while also allowing for the potential discovery of alternative rTCA cycles that run parallel to the primitive cycle. For each off-cycle intermediate, the subset of b2f2 reactions were characterized that converted these species to the next intermediate in the main cycle (*i.e.*, this tested whether the off-cycle intermediates represent a one-reaction detour from the main cycle) or that converted them to the off-cycle intermediates of the next main cycle reaction (*i.e.*, this tested the existence of parallel cycles to the main cycle, blue circles in Fig. 1b). The inclusion of these additional reactions corresponds to the characterization of all cycles that are parallel to the uncatalyzed rTCA cycle but separated by a b2f2 reaction. The topology of the resulting network forms a torus structure around the main rTCA cycle, where each rTCA intermediate can participate in up to two sequential transformations away from the main cycle. This geometric constraint, defined by the b2f2 reaction limitation, represents the minimal complexity needed to evaluate both direct competing pathways and potential alternative cycles. Successfully localized transition states (black lines in Fig. 1b) represent kinetically feasible reactions, while unlocalized transition states (grey lines) indicate mechanistically inaccessible pathways. This pattern of accessible and inaccessible pathways defines the kinetic boundaries of the prebiotic reaction network. An example of a b2f2 exploration about one of the reactants within the main rTCA cycle is shown, where the main target species are highlighted in green, off-target species are marked in red, and potential parallel cycle species are denoted in blue (Fig. 1c).

### 2.3 Comparative analysis of primary mechanisms and off-target pathways

**Fig. 2:**
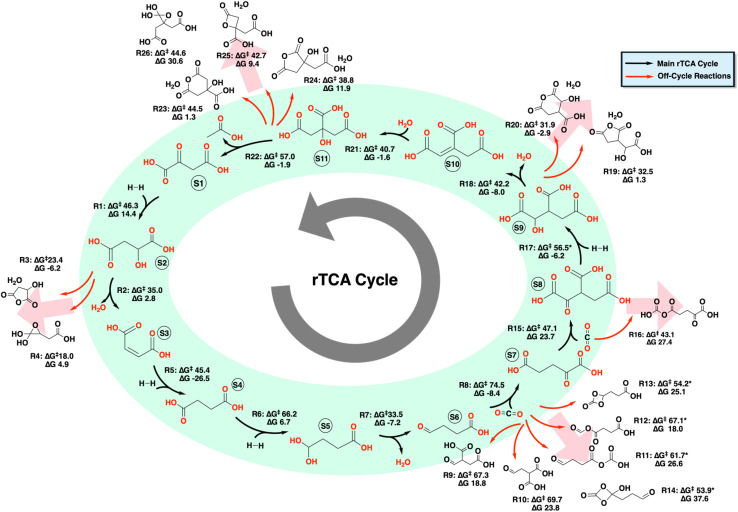
Chemical reaction network (CRN) for the rTCA cycle, generated using the YARP 2.0 methodology. The species numbers S1–S11 indicate known intermediates of the rTCA and the reaction number R1–R26 indicate the reaction in the uncatalyzed rTCA cycle. All the main cycles reactions and the off-cycle reaction with a barrier lower than the corresponding main cycle reactions are show. The * notation indicates barriers without explicit water molecules. The free energies of activation (Δ*G*^‡^) are calculated using both barrier heights with and without an explicit water molecule. The value shown is the lowest of these two mechanisms. The main rTCA cycle is denoted by black arrows. Off-cycle reactions with a free energy of activation (Δ*G*^‡^) lower than that of the main cycle reaction are depicted in red.

The exploration provided a mechanistic and kinetic characterization of the uncatalyzed rTCA cycle and its main off-cycle reactions (Fig. 2). Intended transition states were obtained for all main cycle reactions (see Fig. 4 for main cycle TSs), meaning that the exploration algorithm successfully mapped a continuous path through potential energy space connecting all main cycle intermediates. Notably, TS searches typically fail to converge when applied to unphysical reactions, which supports the basic plausibility of the primitive cycle characterized here.

Activation energies across the cycle range from 33.5 kcal mol^−1^ for the dehydration step in the reductive carboxylation (R7) of succinate (S4) to form 2-oxoglutarate (S7) to 74.5 kcal mol^−1^ for the carboxylation step (R8) in the reductive carboxylation of succinate (S4) to form 2-oxoglutarate (S7). Dehydration reactions (R2, R7, R18) represent the most kinetically accessible transformations, with barriers ranging from 33.5 to 42.2 kcal mol^−1^ (R2, R7, R18). These relatively low barriers suggest dehydration steps could proceed under prebiotic conditions. Although these reactions have a low barrier, the dehydration (R2) of malate (S2) to fumarate (S3) and the dehydration step (R18) in the isomerization of isocitrate (S9) to citrate (S11) have several competing off-target reactions along the main cycle. Three of the rTCA reactions present significant kinetic challenges. Carbonyl reduction of succinate (R6) requires 66.2 kcal mol^−1^, likely due to the formation of a constrained ring transition state. Carboxylation of succinate (R8) presents the highest barrier at 74.5 kcal mol^−1^, reflecting the energetic cost of CO_2_ activation. The C–C bond cleavage of citrate (R22) requires 57.0 kcal mol^−1^, consistent with the challenge of breaking strong carbon–carbon bonds. These high-barrier steps identify specific transformations that would require catalytic assistance for the cycle to operate under prebiotic conditions.

For the main cycle carbonyl reduction and CO_2_ addition reactions, there is potentially some latitude in the specific order and location of the reactions. For example, oxaloacetate (S1) and other intermediates contain multiple distinct carbonyl groups, creating the potential for competing reduction pathways. However, the reduction of the non-target carbonyl groups faces substantially higher barriers. The carboxyl reduction in oxaloacetate (R1) requires 92.7 kcal mol^−1^ compared to the keto group pathway, while competing reductions in R17 face minimum barriers ≥62.7 kcal mol^−1^. These intrinsic reactivity differences naturally enforce the same carbonyl reduction reaction sequences observed in modern enzymatic pathways, suggesting that chemical kinetics may have guided the evolution of the biological rTCA cycle.

**Fig. 3:**
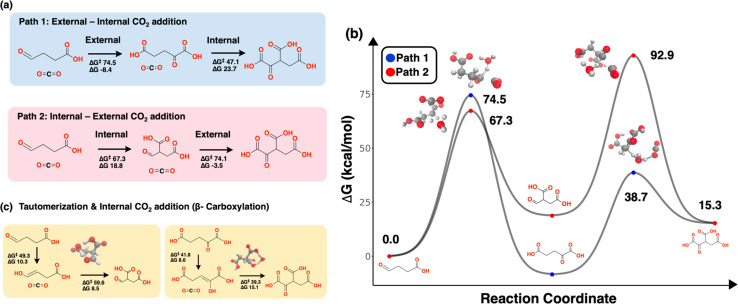
Alternate orderings of CO_2_ additions. (a) Path 1 is the external CO_2_ addition to succinic semialdehyde to form 2-oxoglutarate followed by an external CO_2_ addition to form oxalosuccinic acid. Path 2 is the inverted mechanism where first CO_2_ is added internally to succinic semialdehyde followed by an external addition to form oxalosuccinic acid. (b) The energy landscape of these two pathways. Path 1 is advantageous for the overall cycle. (c) Tautomerization-assisted β-carboxylation *via* b3f3-type pathways proceeds through cyclic transition states involving hydride transfer, either intramolecularly or water-mediated, capturing classical β-carboxylation mechanism.

Likewise, for the CO_2_ additions, two addition orders are possible (Fig. 3). The main cycle mechanism involves an external addition to the aldehyde (R8) followed by an internal addition to the C3 position (R15; Path 1), while the alternative involves a CO_2_ addition to the internal C2 position followed by the aldehyde addition (Path 2). The reaction coordinates for both reaction pathways were localized by the exploration. The internal addition step in Path 2 exhibits a barrier that is 7.2 kcal mol^−1^ lower than the corresponding external addition step in Path 1. However, the second step in Path 2 encounters a steep barrier that is 54.2 kcal mol^−1^ higher. The high barrier steps – specifically the second step in Path 2 and the external addition step in Path 1 – involve complete bond cleavage in the transition state while the internal addition in Path 1 proceeds *via* a concerted mechanism that lowers its barrier (Fig. 2b). These findings underscore that a reduction in the barrier for an initial step does not ensure overall kinetic favorability if a subsequent step is significantly hindered. Overall, the pathway that begins with an external CO_2_ addition followed by an internal addition is kinetically advantageous for the cycle. These reactivity differences again reveal a kinetic preference in the uncatalyzed system that mirrors the rTCA cycle.

An alternative tautomerization-assisted β-carboxylation mechanism was also evaluated, involving the addition of CO_2_ to the β-position of an enolate intermediate (Fig. 3c). This corresponds to a b3f3-type transformation. These β-carboxylation steps proceed through cyclic transition states characterized by hydride transfer from an adjacent hydroxyl group. For succinic semialdehyde (S6)—an analog to the internal carboxylation step in Path 2—the reaction proceeds *via* an intramolecular cyclic hydride transfer without the involvement of water. In contrast, the carboxylation of 2-oxoglutarate (S7)—analogous to the internal addition step in Path 1—requires an explicit water molecule to mediate the hydride transfer through a ring-like transition state. Although these tautomerization-assisted pathways do not offer an energetic advantage, with overall barriers comparable to those of the direct concerted b2f2 mechanisms, they successfully capture the cyclic transition state geometry that is characteristic of β-carboxylation reactions.

Although transition states were localized for all reactions within the uncatalyzed rTCA cycle, these reactions are not all kinetically favored. Competing b2f2 pathways that were explored show lower activation barriers than many of the main cycle reactions. The dehydration of malate (S2) that yields fumarate (S3) has several competing reaction channels with barriers of 23.4 kcal mol^−1^ (R3) and 18.0 kcal mol^−1^ (R4), substantially lower than the main cycle barrier of 35.0 kcal mol^−1^ (R2). This pattern continues throughout the cycle. In the conversion of succinate (S4) to 2-oxoglutarate (S7), the carboxylation step (R8) competes with alternative pathways, showing barriers as low as 53.9 kcal mol^−1^ (R14). Similarly, the carboxylation of 2-oxoglutarate (S7) to isocitrate (S9) faces competing pathways with barriers as low as 43.1 kcal mol^−1^ (R16). The isomerization of isocitrate (S9) to citrate (S11) proceeds through dehydration and hydration steps, where the dehydration step (R18) with a barrier of 42.2 kcal mol^−1^ competes with pathways as low as 31.9 kcal mol^−1^ (R20). Finally, the bond cleavage of citrate (S11) to form acetate and oxaloacetate (S1) encounters competing channels with barriers down to 38.8 kcal mol^−1^ (R24). These systematically lower barriers for competing pathways reveal fundamental kinetic limitations in the uncatalyzed cycle.

We investigated whether parallel reaction cycles might provide alternative pathways around the kinetic bottlenecks in the main rTCA cycle. The reaction exploration algorithm created two possibilities for discovering alternatives. First, reactions that might return off-cycle products to the main cycle intermediates. Second, reactions that could connect sequential off-cycle products into alternative cycles. Both possibilities proved mechanistically unfeasible with the side products that were discovered. Note that only a subset of the reactions that are characterized converge to an intended TS. Out of the subset of side-products with intended TS, none could reconnect with the main cycle or another side-product through b2f2 reactions. The nearest connections possible required the simultaneous breaking and forming of four chemical bonds. Such complex transformations lie in energetically unfavorable regions of the potential energy surface, making TS localization computationally intractable. A complete list of the reaction and kinetic parameters is presented in Table S1 (SI Section 2). This finding suggests that kinetics more tightly circumscribe the range of parallel and partially overlapping cycles than is suggested by earlier studies that only considered the thermodynamics of alternative cycles.

The main cycle reactions are generally thermodynamically favorable or nearly thermoneutral, but several key steps exhibit prohibitively high activation barriers (*e.g.*, succinic semialdehyde carboxylation, Δ*G*^‡^ = 74.5 kcal mol^−1^; isocitrate reduction, Δ*G*^‡^ = 56.5 kcal mol^−1^), rendering them kinetically inaccessible under mild prebiotic conditions. In contrast, numerous off-cycle reactions proceed through lower-barrier pathways, diverting flux away from productive carbon fixation. These side reactions yield off-target products and undermine the continuity of the main cycle. The prevalence of thermodynamically favored or neutral yet kinetically unfavorable main cycle routes & the presence of thermodynamically and kinetically favorable off-target routes underscore the dominance of kinetic control in this reaction network and further illustrate the necessity of catalysis to suppress parasitic pathways and enable sustained turnover of the rTCA cycle.

**Fig. 4:**
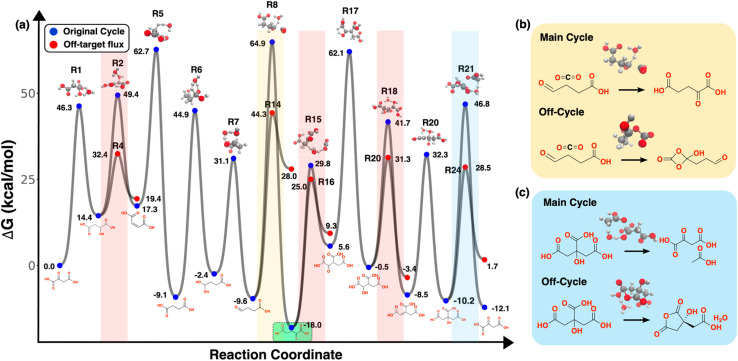
Free energy landscape of the uncatalyzed rTCA cycle generated using YARP methodology. (a) The curve with blue markers represents the primary rTCA cycle, while the broken curves with red markers depict kinetically competing pathways with the lowest free energies of activation. The top row of molecular structures displays the transition state geometries for the main rTCA pathway, and the bottom row presents key ground state components of the main rTCA pathway. Shaded regions indicate areas where off-cycle flux occurs leading to kinetic bottlenecks. (b) and (c) offer a more detailed view of the main rTCA and off-cycle reaction transition states for two specific cases, with shaded regions color-coordinated to facilitate comparison.

Analysis of the energy diagram for the uncatalyzed rTCA cycle demonstrates that while the pathway satisfies the thermodynamic requirements for carbon fixation, it fails to meet the necessary kinetic criteria for spontaneity (Fig. 4). The overall cycle shows clear thermodynamic favorability with a net free energy change of −12.14 kcal mol^−1^ for converting two CO_2_ and four H_2_ molecules to acetate and water. This value aligns with thermodynamic data reported in previous studies on the reductive tricarboxylic acid (rTCA) cycle, and aligns with the thermodynamics of formation of acetic acid from CO_2_ and H_2_.^18,19^ However, examination of the complete energy landscape reveals kinetic barriers that prevent this thermodynamically favorable conversion. Five main cycle reactions exhibit at least one side reaction that is kinetically favored despite being more endothermic than the main cycle reaction. Two representative cases are highlighted in Fig. 4b and c with their transition states and competing reactions. The carboxylation of succinate to 2-oxoglutarate competes with a kinetically favorable CO_2_ addition that is geometrically favored (the TS is a direct adduct to the carboxylic acid) but forms a relatively unstable four-membered ring (Fig. 4b). Similarly, the cleavage of citrate to acetate and oxaloacetate competes with an intramolecular isomerization that is kinetically favored by 18.2 kcal mol^−1^ (Fig. 4c). These off-target fluxes are expected to be irreversible to the extent that these species undergo additional kinetically favored reactions besides the reverse reaction. The energy diagram also reveals that the main cycle intermediate, 2-oxoglutarate (S7), is a local thermodynamic sink in the cycle that resides ∼6 kcal mol^−1^ lower in energy than the globally favored production of acetic acid (Fig. 3a, green). In kinetic terms, this means that the second CO_2_ addition and other steps downstream of 2-oxoglutarate must compete with their reverse reactions, which locally favor 2-oxoglutarate accumulation. This may be within the error of the DFT level of theory and PCM model used here; nevertheless, it is indicative of the finely balanced energetics of the rTCA cycle and shows how the thermodynamics of intermediates can also modulate the overall cycle kinetics.

While the b2f2 rule defines the extent of topological modification explored, the resulting transition states reveal several known mechanistic reaction pathways (Fig. 4a). The TS for R2 and R18 display features characteristic of an E1-type elimination mechanism, where the β-hydrogen is fully abstracted in the transition state, resulting in the formation of a polar intermediate. Similarly, in reaction R22, the transition state (Fig. 4a) shows C–C bond cleavage occurring concurrently with proton abstraction from the adjacent hydroxyl group, facilitated by water, consistent with a retro-aldol elimination mechanism. These cases demonstrate that, although the reaction network was generated using a mechanism-agnostic b2f2 rule, realistic mechanistic features, including stepwise proton transfers and charge-separated intermediates, emerge naturally during transition state optimization. Additionally, two pathways that generate species known to exist in proximity to the rTCA cycle chemical space, such as glyoxylic acid, formic acid, and pyruvic acid, are presented in the SI Section 4.^3,5,20^

### 2.4 Microkinetic modelling of uncatalyzed rTCA cycle

**Fig. 5:**
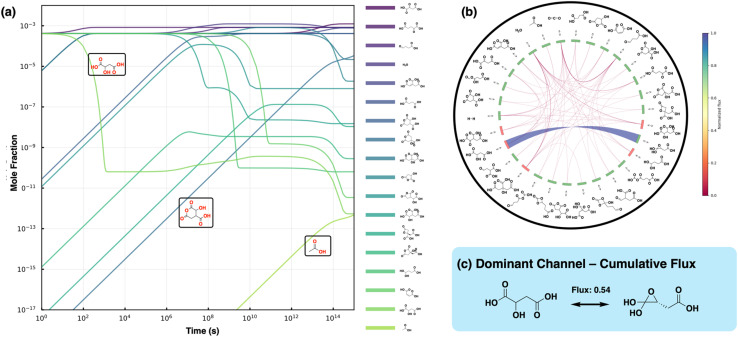
Kinetic analysis of the uncatalyzed rTCA cycle. (a) Microkinetic modelling of the uncatalyzed rTCA cycle. The plot shows only those species with a final mole fraction greater than 10^−14^. The curves are colored as per their final mole fractions. Curves corresponding to oxalosuccinic acid, 3-hydroxy-2,6-dioxooxane-4-carboxylic and acetate. (b) Visualization of the cumulative flux analysis of the whole network. Each link connecting the molecular species corresponds to a reaction involving those species and the width and the color (shown in the color bar) of the link correspond to the normalized flux. The color of the label corresponds to the net inward flux (green) or net outward flux (red) for the respective molecular species. (c) The reaction channel with the most dominant cumulative fluxes. The value of the cumulative fluxes is given above the arrow.

To evaluate the kinetic feasibility of the uncatalyzed rTCA cycle under prebiotic conditions, we performed microkinetic modeling simulations of the discovered network (Fig. 5). Microkinetic modeling simulations were performed at 348.15 K and 1 atm pressure with excess H_2_ and CO_2,_ which reflect favorable conditions for promoting carbon fixation (*i.e.*, hot and with ample H_2_ as fuel).^21^ For each forward reaction, the corresponding reverse reaction is included using barriers derived from the computed activation-free energies (Δ*G*^‡^) and reaction-free energies (Δ*G*). Cumulative flux represents the total mass flow through a reaction channel, calculated as the absolute sum of forward and reverse fluxes. It serves as a measure of overall channel utilization, highlighting high-traffic pathways regardless of direction. In contrast, net flux is defined as the forward minus reverse flux and indicates the net directional flow of material, indicating whether species are being accumulated or depleted.

The system was initialized uniformly with a mole fraction of 0.0004 for all main rTCA cycle species while maintaining excess CO_2_ and H_2_ with mole fractions of 0.82 and 0.16, respectively. Although CO_2_ and H_2_ have different aqueous solubilities (CO_2_ being orders of magnitude greater than H_2_), the simulations assume their sustained availability to probe the intrinsic reaction kinetics. All reactions in the microkinetic simulations are treated as elementary and reversible, consistent with transition state theory. Under these conditions, a kinetically viable cycle would show significant fluxes through all participating species with monotonic accumulation of acetic acid. In contrast, the microkinetic simulations show an accumulation of the main cycle species 2-oxoglutarate (S7) and oxaloacetate (S1), which is consistent with their thermodynamic minima along the main cycle (Fig. 5a). Cumulative main cycle flux is also lost to the low barrier off-target reaction, R4, of 0.54 mol m^−2^ s^−1^ (Fig. 5b and c). In terms of net flux (forward–backward), the reverse reaction of R15 has the maximum net flux of 8.4 × 10^−5^ mol m^−2^ s^−1^, followed by the forward reaction of R20 with a net flux of 4.3 × 10^−5^ mol m^−2^ s^−1^. This is also evident in the depletion of oxalosuccinic acid and accumulation of 3-hydroxy-2,6-dioxooxane-4-carboxylic acid (highlighted in Fig. 5a). Despite 2-oxoglutarate (S7) being a local energetic sink, completion of the cycle is energetically favorable as the free energy gain is directly proportional to the number of cycles completed. If the cycle terminates at 2-oxoglutarate (S7), there will be a one-time free energy gain of −18 kcal mol^−1^; however, completion of the cycle provides −12.1 kcal mol^−1^ free energy gain per cycle, which will grow linearly with the number of cycles. The microkinetic simulations indeed recapitulate the monotonic increase in acetic acid (highlighted in Fig. 5a), but it is extremely small (5 × 10^−13^) despite the long timescales that were simulated due to the presence of a kinetic sink in 2-oxoglutarate and high barriers throughout the cycle. This pronounced flux localization demonstrates that even under favorable conditions with excess reactants, the uncatalyzed cycle cannot sustain the distributed reaction flux necessary for effective carbon fixation.

Several of the off-cycle intermediates correspond to cyclic anhydrides formed through intramolecular dehydration of dicarboxylic or hydroxy-dicarboxylic acids. 3-Hydroxy-2,6-dioxooxane-4-carboxylic acid, which accumulates through reaction (R20), is an anhydride derivative of isocitric acid. Reaction (R24) likewise produces citric anhydride, consistent with experimental observations of its formation under mild dehydrating conditions.^22^ The emergence of anhydrides in our network reflects their intrinsic kinetic favorability, as dicarboxylic acids readily undergo intramolecular cyclization to form stable five-membered anhydrides.^23^ Moreover, such anhydride intermediates have been experimentally identified in studies of prebiotic analogues of the rTCA cycle, where they function as precursor species enabling thioester formation.^24^

The coupled pericycle connecting acetate to oxaloacetate has been proposed as a route by which the rTCA cycle becomes autocatalytic.^18^ Our analysis of this pericycle indicates that the key carboxylation steps exhibit activation barriers comparable to those in the main rTCA cycle. Consequently, inclusion of this auxiliary pathway does not significantly enhance the overall kinetic feasibility or alleviate the primary rate-limiting steps. This analysis is discussed in detail in SI Section 5.

## 3 Conclusion

This study presents a kinetic characterization and analysis of the uncatalyzed rTCA cycle using recently developed reaction network exploration methods. The resulting network comprises hundreds of quantum chemically characterized reactions, providing a complete kinetic description of the uncatalyzed cycle and its side reactions. The results demonstrate that kinetic constraints severely restrict cycle feasibility under uncatalyzed prebiotic conditions, despite thermodynamic calculations suggesting the viability of both the primary cycle and parallel alternatives. Several reaction sequences in the uncatalyzed cycle show kinetic preferences matching modern enzymatic pathways, particularly in the ordering of CO_2_ additions and carbonyl reductions, even though alternative sequences are thermodynamically possible. Nevertheless, kinetically favored side reactions were identified for five of the main cycle reactions, and no kinetically competitive parallel cycles were discovered. Under the simulated conditions, the primitive rTCA cycle is also kinetically inhibited by the relatively stable 2-oxoglutarate intermediate that forms after the first CO_2_ addition and acts as a local thermodynamic sink. These findings reveal how kinetic factors constrained the emergence of the modern rTCA cycle and highlight several species whose conversion would have to be catalyzed in prebiotic predecessors of the rTCA cycle.

Our study highlights the kinetic bottlenecks associated with the rTCA cycle under prebiotic conditions. This kinetic limitation implies that the emergence of catalytic strategies would have been essential for enabling sustained flux through the cycle and hence the widespread conservation of the rTCA cycle across diverse forms of life. The evolutionary retention of the rTCA cycle may therefore reflect its compatibility with catalytic enhancement, rather than intrinsic kinetic accessibility in its uncatalyzed form. Specifically, the cycle's modular structure may have made it particularly amenable to stepwise recruitment of catalysts that overcame otherwise prohibitively high barriers.^25^ Notably, the absence of plausible parallel cycles in our analysis further highlights the unique adaptability of the rTCA cycle to catalytic reinforcement. Thus, our results support the view that kinetic constraints shaped early metabolic evolution and suggest that the prevalence of the rTCA cycle may stem not from its feasibility, but from its evolutionary adaptability once catalysis became available. These findings are consistent with the idea that early carbon fixation cycles may have relied on other mechanisms to facilitate chemical reactions in a prebiotic environment or in combination with the compartmentalization of certain reactions within the rTCA cycle, catalyzed or as shown in several studies that use Zn, Cr, or Fe as potential catalysts.^5,20,26^

Several methodological limitations should be considered when interpreting these results. The quantum chemical calculations underlying our kinetic predictions carry inherent uncertainties from the use of PCM solvation models, DFT-level electronic structure methods, and finite conformational sampling. Recent benchmarking studies in related reaction networks suggest these approximations introduce uncertainties of 3–6 kcal mol^−1^ in computed barrier heights and reaction energies.^27–29^ While these uncertainties affect quantitative comparisons between specific reaction pathways, the qualitative conclusions about reaction preferences remain robust, given the larger energy differences observed between competing channels. A more fundamental limitation arises from restricting reaction exploration to b2f2 mechanisms within one step of the main cycle. More complex reaction sequences involving three-bond changes or multiple steps could potentially enable parallel cycles that bypass the identified kinetic bottlenecks. The water-catalyzed mechanisms investigated here also represent a minimal model of solution-phase catalysis, as certain reactions might proceed through mechanisms involving multiple water molecules or alternative catalytic species.^30,31^ Further, the absence of a converged transition state does not prove that no such reaction is possible. While YARP's transition state localization strategy has consistently localized TSs across a broad range of chemistries (*e.g.*, identifying lower-barrier TSs for known reactions and uncovering TSs in previously unresolved or unexplored pathways), the future discovery of a “missed reaction” involving the rTCA main cycle species cannot be ruled out.^32^

The systematic computational approach developed in this work enables broader investigations of prebiotic carbon-fixation chemistry. This methodology can be directly applied to analyze other anaerobic carbon fixation cycles proposed to operate under early Earth conditions, to interrogate their kinetic viability and catalytic requirements. The reaction network exploration strategy demonstrated here can be accelerated through machine learning assistance in TS searches and reaction pathway prediction, enabling more exhaustive mapping of the chemical space around carbon fixation cycles. Such enhanced sampling could support the discovery of *de novo* carbon fixation cycles optimized for specific criteria, such as kinetic efficiency or minimal catalytic requirements. The resulting fixation cycles could provide new hypotheses for prebiotic chemistry while suggesting efficient chemical pathways for carbon dioxide reduction and sequestration. Identifying kinetically viable carbon fixation cycles would have implications for both origins of life research and the development of new technologies for addressing atmospheric carbon dioxide accumulation.

## 4. Computational methods

### 4.1 Reaction mechanism generation

The Yet Another Reaction Program (YARP 2.0) was utilized to study the kinetics of the uncatalyzed rTCA cycle and competing side reactions involving its intermediates.^12,33^ YARP methodology enumerates the reactions by generic graphical rules to find the potential products with the given reactants. These rules take the form of break *n* bonds and form *m* bonds (b*n*f*m*), where *n* and *m* can vary based on the reaction template. YARP uses these graphical rules to generate potential products and then a double-ended transition state search to localize and compare transition states of competing reactions. In this study, the b2f2 reaction enumeration rules were applied to enumerate possible reactions, and the rTCA cycle was decomposed into several elementary reaction steps that conform to the b2f2 reaction rule. To localize the TS of the reaction channel connecting off-cycle species back into the main cycle and the reaction channel connecting two off-cycle species to form parallel cycles adjacent to the main rTCA cycle. The enumeration step was circumvented and established the reactant–product pairs by utilizing the geometries of the off-cycle product and the main cycle intermediate or the two off-cycle species. Ten distinct reactant–product conformer pairs are established using conformational sampling methods implemented in the CREST package.^34^ Transition states were located by employing the Growing String Method (GSM), followed by Berry quasi-Newton optimization and verification through Intrinsic Reaction Coordinate (IRC) calculations. Initial GSM searches and node optimizations used a tolerance of 0.01 hartree Bohr^−1^ with 11 images per reaction path. IRC calculations were performed using 60 points with a step size of 5 Bohr. The initial searches were conducted at the GFN2-xTB level of theory, while transition state refinements employed the ωB97X-D functional with the def2-TZVP basis set and a polarizable continuum model (PCM) with a dielectric constant of *ε* = 78.4 using a superfine integration grid.^35,36^ Two sets of calculations were performed, one with just the implicit solvation model of water to account for the solvation effects of the aqueous environment of the biochemical reaction, and the other was the water-catalyzed mechanism. The water-catalyzed reaction mechanism refers to the additional transfer of one or several protons to or from water molecules in the TS and the subsequent alteration of the Potential Energy Surface (PES). The water-catalyzed reaction mechanism is generated by using the graphical enumeration described previously. Subsequently, a water molecule is added in the spatial vicinity of reactive centers in the spatial coordinate files of the reactant–product pairs, to account for the effect of water molecules being present in the solution and their interaction with the reactants and products. All DFT calculations were carried out by Gaussian 16.^37^ All GFN2-xTB calculations were performed with the xTB program (version 6.4.0).^33^

### 4.2 Kinetic modeling

All kinetic modeling was performed using the Cantera software package.^38^ All reactions were assumed to be elementary, with rates being calculated according to transition-state theory (TST). In our study, entropy corrections were performed to all DFT-calculated transition-state energies to get the Gibbs free energies, following the thermochemistry methodology implemented in Gaussian 16.^37^ The parameters in Cantera were calculated based on the YARP-calculated free energies of activation, Δ*G*^‡^, for each reaction and the modified Arrhenius equation.^39^1
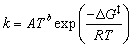
2
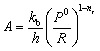

3 *b* = *n*_r_

where *k*_b_, *h*, and *R* are the Boltzmann, Planck, and ideal gas constants, respectively, and *P*^0^ and *n*_r_ are the standard atmosphere pressure and number of reactants, respectively. During the kinetic simulations, the mole fraction of each species was obtained by the states class in Cantera, while the cumulative net flux was computed by summing up the net flux of each time step from the net rates of progress function in Cantera.

## Author contributions

V. S., J. M, and B. M. S. conceived and designed the study. V. S. generated and analyzed the data and wrote the paper. J. M. oversaw the project and wrote the paper. B. M. S. oversaw the project and wrote the paper.

## Conflicts of interest

The authors declare no conflict of interest.

## Supplementary Material

SC-OLF-D5SC05872D-s001

SC-OLF-D5SC05872D-s002

## Data Availability

The authors declare that the data supporting the findings of this study are available within the paper and its supplementary information (SI). The version of YARP (v2.0) and the reaction conformational sampling package used in this study are available through GitHub under the GNU GPL-3.0 License [https://github.com/Savoie-Research-Group/yarp]. Supplementary information: additional reaction comparisons and technical details referenced in the main text. See DOI: https://doi.org/10.1039/d5sc05872d.
