# Supplementary material for: Kinetics overcome thermodynamics in primitive analogs of the reverse tricarboxylic acid cycle
Source: Chem Sci. 2026 Feb 4;17(12):6197–207. doi: 10.1039/d5sc05872d (PMC12869705; doi:10.1039/d5sc05872d)
Supplement: SC-017-D5SC05872D-s002 [file SC-017-D5SC05872D-s002.pdf]

Supplementary Information (SI) for Chemical Science.
This journal is © The Royal Society of Chemistry 2025

Supporting Information: Kinetics Overcome Thermodynamics in Primitive Analogs of the Reverse Tricarboxylic Acid Cycle

Vignesh Sathyasaleen[†], John Morgan[†]*, Brett M. Savoie*[†‡]

** Corresponding Authors:* jamorgan@purdue.edu, bsavoie2@nd.edu

[†]Davidson School of Chemical Engineering, Purdue University, West Lafayette, Indiana 47906, United States

[‡]Department of Chemical and Biomolecular Engineering, University of Notre Dame, Notre Dame, Indiana 46556, United States

1. Effect of Explicit Water on Reaction Energetics:

The presence of water molecules surrounding the reaction center can modulate reaction dynamics by altering hydrogen-bonding patterns and stabilizing transition states, thereby influencing both the energy profiles and activation barriers. To quantitatively evaluate these effects, explicit water molecules were positioned in the spatial vicinity of the reactive centers in the coordinate files of each reactant–product pair. Incorporation of a single water molecule into the reaction environment led to an average reduction in activation barrier of 9.5 $kcal.mol^{-1}$, with approximately 77 % of all reactions exhibiting lower barriers compared to their uncatalyzed counterparts (Fig. S1). Analysis by mechanistic class revealed that Cleavage (–13.7 $kcal.mol^{-1}$), Hydrolysis (–12.8 $kcal.mol^{-1}$), Dehydration (–12.4 $kcal.mol^{-1}$), and Hydrogenation (–9.0 $kcal.mol^{-1}$) reactions showed the largest catalytic enhancement, consistent with water's ability to mediate proton transfer in transition states. Cyclization (–7.0 $kcal.mol^{-1}$) and Carboxylation (–4.8 $kcal.mol^{-1}$) reactions were less affected. This underscores the crucial role that water plays in facilitating biochemical reactions, and this has been observed in other studies. (1)

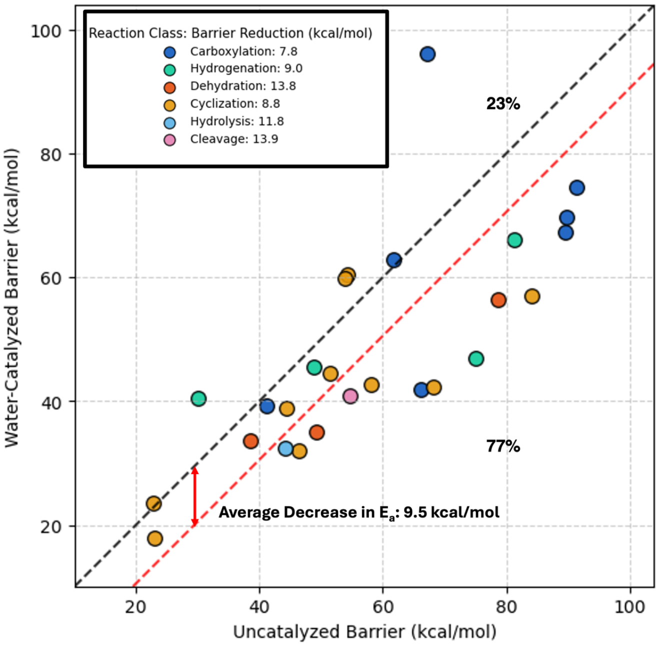

Figure S1: Comparison of Non-catalyzed vs Water Catalyzed barriers. The water molecule present in the vicinity of the reaction centers enables the transfer of one or several protons to or from water molecules in the TS and has a significant impact on the reaction energetics and decreases the barriers on an average by 9.5 kcal.mol^{-1}.

2. Kinetic Data:

Table S1 presents the SMILES representations of the reactions, along with their corresponding free energies of activation and free energy values, calculated at a reference temperature of 298.15 K, presented in the main text Figure 2. These kinetic parameters were employed for the kinetic analysis. The computational cost of the simulations was assessed by tracking the number of DFT-level gradient calls (GCs). (2) Notably, while direct comparisons are challenging due to differences in the level of theory and reporting, AIMD simulations typically require approximately 10 picoseconds to converge reactions, resulting in roughly 10^4 DFT calls. (3) In contrast, our

YARP-based approach can achieve convergence with two orders of magnitude fewer computationally expensive DFT gradient calls. This significant reduction in computational cost underscores the efficiency of the YARP method, making it a valuable tool for exploring complex biochemical reactions.

Table S1: Reactions and Kinetic Parameters

**S.No**	**Reaction Smiles**	$\Delta G^{\ddagger}$ **kcal.mol^{-1}**	ΔG **kcal.mol^{-1}**	**Gradient Calls**
1	O=C(C(CC(O)=O)=O)O.[H][H]>>OC(C[C@@H](O)C(O)=O)=O	46.3	14.4	8
2	OC(C[C@@H](O)C(O)=O)=O>>OC(/C=C\C(O)=O)=O.O	35.0	2.8	12
3	O=C(C(CC(O)=O)=O)O.[H][H]>>O=C1C[C@@H](O)C(O1)=O.O	23.4	-6.2	11
4	OC(C[C@H]1OC1(O)O)=O>>OC(C[C@@H](O)C(O)=O)=O	18.0	4.9	21
5	OC(/C=C\C(O)=O)=O.[H][H]>>OC(CCC(O)=O)=O	45.4	-26.5	6
6	OC(CCC(O)=O)=O.[H][H]>>OC(CCC(O)=O)O	66.2	6.7	7
7	OC(CCC(O)=O)O>>O=CCCC(O)=O.O	33.5	-7.2	6
8	O=CCCC(O)=O.O=C=O>>OC(CCC(C(O)=O)=O)=O	74.5	-8.4	11
9	O=CCCC(O)=O.O=C=O>>O=C[C@H](CC(O)=O)C(O)=O	67.3	18.3	28
10	O=CCCC(O)=O.O=C=O>>O=CCC(C(O)=O)C(O)=O	69.7	23.8	31
11	O=CCCC(O)=O.O=C=O>>O=CCCC(OC(O)=O)=O	61.7	26.6	7
12	O=CCCC(O)=O.O=C=O>>O=COC(CCC(O)=O)=O	67.1	18.0	6
13	O=CCCC(O)=O.O=C=O>>OC(CCC1OC(O1)=O)=O	54.2	25.1	6
14	O=CCCC(O)=O.O=C=O>>O=CCCC1(OC(O1)=O)O	53.9	37.6	8
15	OC(CCC(C(O)=O)=O)=O.O=C=O>>OC(CC(C(C(O)=O)=O)C(O)=O)=O	47.1	23.7	70
16	OC(C/C=C(C(O)=O)\O)=O.O=C=O>>OC(OC(CCC(C(O)=O)=O)=O)=O	43.1	27.4	16

17	OC(C[C@H](C(C(O)=O)=O)C(O)=O)=O.[H][H]>>OC(C[C@@H](C(O)=O)[C@@H](O)C(O)=O)=O	56.5	-6.2	8
18	OC(C[C@@H](C(O)=O)[C@@H](O)C(O)=O)=O>>OC(C/C(C(O)=O)=C/C(O)=O)=O.O	42.2	-8.0	8
19	OC(C[C@@H](C(O)=O)[C@@H](O)C(O)=O)=O>>O=C1OC([C@H]([C@H](O)C(O)=O)C1)=O.O	32.5	1.3	11
20	OC(C[C@@H](C(O)=O)[C@@H](O)C(O)=O)=O>>O=C2OC([C@@H](O)[C@@H](C(O)=O)C2)=O.O	31.9	-2.9	11
21	OC(C/C(C(O)=O)=C/C(O)=O)=O.O>>OC(C(O)(CC(O)=O)CC(O)=O)=O	40.7	-1.6	7
22	OC(C(O)(CC(O)=O)CC(O)=O)=O>>O=C(C(CC(O)=O)=O)O.CC(O)=O	57.0	-1.9	8
23	OC(C(O)(CC(O)=O)CC(O)=O)=O>>O=C1OC(CC(O)(C(O)=O)C1)=O.O	44.5	1.3	11
24	OC(C(O)(CC(O)=O)CC(O)=O)=O>>OC(C[C@]1(CC(OC1=O)=O)O)=O.O	38.8	11.9	16
25	OC(C(O)(CC(O)=O)CC(O)=O)=O>>OC(C[C@]1(C(O)=O)CC(O1)=O)=O.O	42.7	9.4	12
26	OC(C(O)(CC(O)=O)CC(O)=O)=O>>OC(CC1(OC1(O)O)CC(O)=O)=O	48.6	30.6	32

3. High pH Reaction Network:

All species within the main rTCA cycle are mild to strong Brønsted acids, with pKa values ranging from 2 to 6, and are therefore expected to exist predominantly in their deprotonated forms under high-pH aqueous conditions. (4) Transition state optimization was conducted on reactions within this deprotonated network to evaluate kinetic accessibility. Despite extensive search efforts, only one transition state was discovered, corresponding to the hydrogenation of 2-oxoglutarate to succinate. While this reaction is thermodynamically favorable ($\Delta G = -23.1$ kcal.mol^{-1}), it proceeds

through a high-energy transition state with an activation barrier of $\Delta G^{\ddagger} = 55.2$ kcal.mol^{-1}. Notably, the activation barrier is comparable to that of the non-deprotonated analog shown in Fig. 2, suggesting that deprotonation has minimal impact when the charged moieties are spatially distal from the reaction center. This is consistent with the expectation that reactions involving direct participation of deprotonated carboxyl groups will be more sensitive to charge effects, whereas deprotonated sites separated by two or more bonds from the reactive center exert a smaller influence on the barrier. (5)

No other anionic transition states were converged for other steps within the main cycle, precluding a complete kinetic comparison and limiting mechanistic insight across the network. These failures are likely due to limitations of the GFN2-xTB method employed during the low-level search phase. (6) This method uses a multipole expansion to approximate electrostatic interactions, which inadequately stabilizes highly charged anions, resulting in poor convergence behavior. Over 200 conformers were sampled in these cases, more than the ~10 used for other reactions, yet no transition states were located. Future efforts will explore alternative levels of theory during the initial transition state localization to improve convergence rates for reactions involving highly charged intermediates, which has shown success in other systems. (7)

Reactant	Product	TS Geometry	TS Geometry (+H2O)	Barrier (kcal/mol)
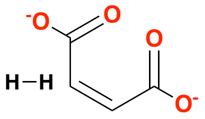	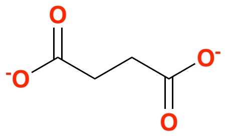	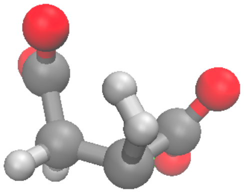	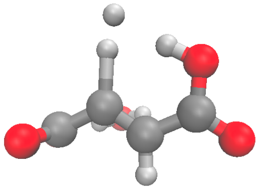	ΔG‡ 55.2<br>ΔG -23.1

Figure S2: Hydrogenation of 2-oxoglutarate to succinate in the doubly deprotonated state proceeds through a high-energy transition state (ΔG‡ = 55.2 kcal.mol^{-1}) despite a favorable overall reaction free energy (ΔG = –23.1 kcal.mol^{-1}). Transition state geometries are shown both in the absence and presence of an explicit water molecule.

4. Additional Reactions of Interest:

Two additional pathways converged that highlight the formation of intermediates chemically adjacent to the reverse tricarboxylic acid (rTCA) cycle (Fig. S3). Reaction 1 yields glyoxylic acid and oxalate from isocitrate via a concerted bond rearrangement, with an activation barrier (ΔG‡) of 78.0 kcal.mol^{-1} and a moderately free energy change (ΔG) of –5.3 kcal.mol^{-1}. Reaction 2 forms pyruvic acid and formic acid from glycolic acid and acetic acid. The reaction proceeded through a higher energy-constrained ring-like transition state with a barrier of 93.0 kcal.mol^{-1} and a reaction-free energy of –3.9 kcal.mol^{-1}. These reactions highlight kinetically accessible known rTCA-adjacent metabolites. (8–10)

5.

Reaction	Reactant	Product	TS Geometry	Barrier (kcal/mol)
1	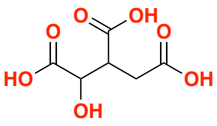	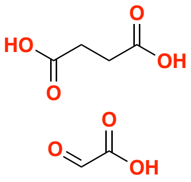	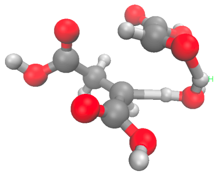	$\Delta G^{\ddagger}$ 78.0<br>ΔG -5.3
2	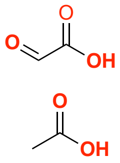	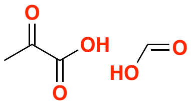	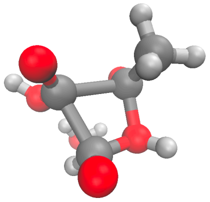	$\Delta G^{\ddagger}$ 93.0<br>ΔG -3.9

Figure S3: Two reaction pathways 1 and 2 that generate species known to exist adjacent to the rTCA cycle chemical space, such as glyoxylic acid, formic acid, and pyruvic acid.

5. Autocatalytic effect:

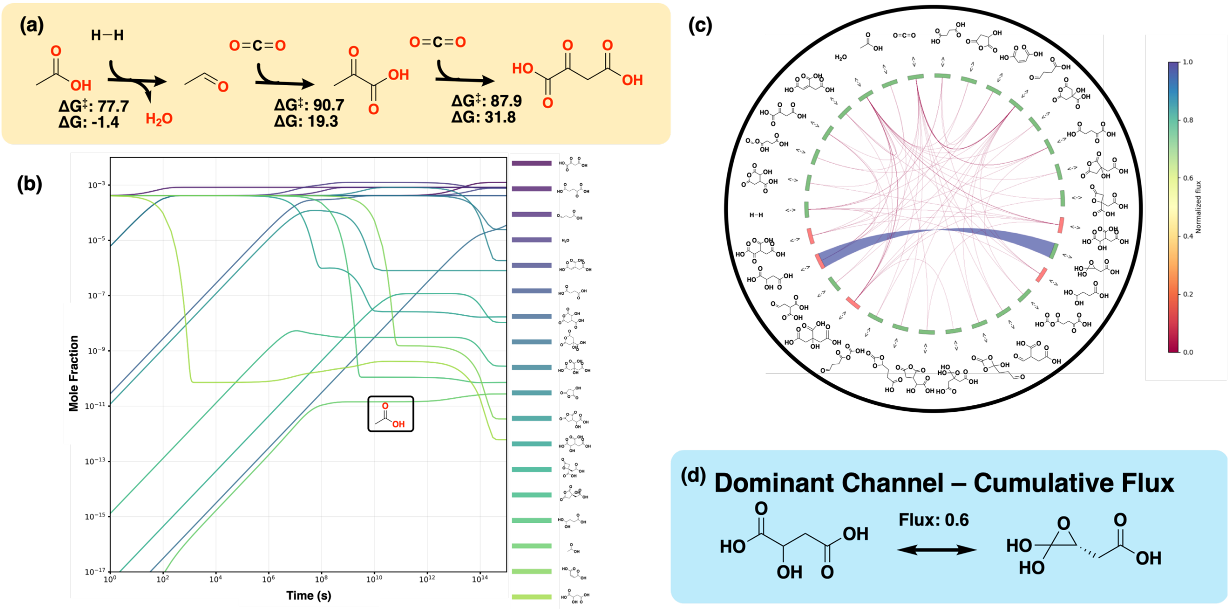

Figure S4: Autocatalytic extension of the rTCA cycle through the acetic acid–oxaloacetic acid pericycle. (a) The elementary reaction steps leading to oxaloacetic acid formation from acetic acid, with calculated reaction free energies and activation barriers. (b) Presents the microkinetic simulations of the rTCA cycle including the pericycle. Despite the high kinetic barrier of the epicyclic pathway rendering it kinetically hindered, this route was incorporated into the model using the lowest-barrier reaction identified from the main rTCA cycle ($\Delta G = -7.0$ kcal.mol^{-1}, $\Delta G^{\ddagger} = 33.0$ kcal.mol^{-1}), allowing assessment of its potential contribution to overall network flux. (c) Visualization of the cumulative flux analysis of the whole network. Each link connecting the molecular species corresponds to a reaction involving those species and the width and the color (shown in the color bar) of the link correspond to the normalized flux. The color of the label corresponds to the net inward flux (green) or net outward flux (red) for the respective molecular species. (d) The reaction channel with the most dominant cumulative fluxes. The value of the cumulative fluxes is given above the arrow.

Three additional reactions were identified that form the basis of the acetic acid–oxaloacetic acid pericycle, an autocatalytic extension of the reverse tricarboxylic acid (rTCA) cycle (Fig. S4a; TS geometries are shown in Fig. S5). (11) Reaction 1 involves the hydrogenation of acetic acid to acetaldehyde and water, proceeding with an activation barrier ($\Delta G^{\ddagger}$) of 77.7 kcal.mol^{-1} and a

reaction free energy (ΔG) of −1.4 $kcal.mol^{-1}$. Reaction 2 corresponds to the carboxylation of acetaldehyde to pyruvic acid, with a higher barrier of 90.7 $kcal.mol^{-1}$ and ΔG of 19.3 $kcal.mol^{-1}$ Reaction 3 involves the subsequent carboxylation of pyruvic acid to form oxaloacetic acid, with $\Delta G^{\ddagger}$ = 87.9 $kcal.mol^{-1}$ and ΔG = 31.8 $kcal.mol^{-1}$. The large activation barriers indicate that the autocatalytic route remains kinetically hindered under uncatalyzed prebiotic conditions.

Despite the high activation barriers rendering the autocatalytic pathway kinetically unfavorable, the potential effect of autocatalysis was examined by incorporating this chemical transformation by modifying the barriers to match the lowest-barrier reaction from the main rTCA cycle ($\Delta G^{\ddagger}$ = 33.5 $kcal.mol^{-1}$, corresponding to R7 in main text Fig. 2) into the microkinetic model (Fig. S4b). The same microkinetic modeling methodology as the main text analysis was followed here. Even with this substitution, the cycle remained kinetically hindered, showing limited enhancement in overall turnover. Cumulative flux analysis revealed that the main cycle flux was partially diverted to an off-target low-barrier reaction, R4, with a flux magnitude of 0.6 $mol.m^{-2}.s^{-1}$ (Fig. S4c-d). Acetic acid formation was notably accelerated, becoming observable in the microkinetic modelling as early as 10^4 s, in contrast to 10^{10} s in the non-autocatalytic simulations presented in the main text. Thus, even if the autocatalytic route were kinetically accessible, it would only marginally enhance the formation of specific intermediates and would not overcome the kinetic bottlenecks that constrain the rTCA cycle under uncatalyzed prebiotic conditions.

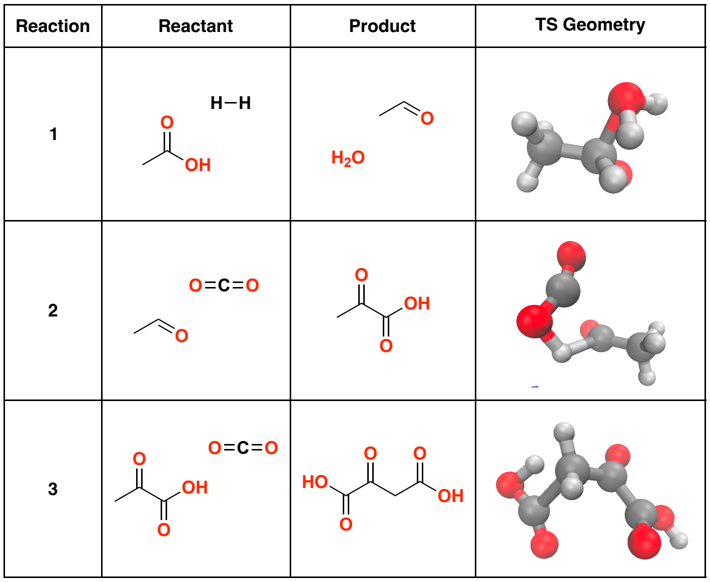

Figure S5: Transition-state geometries of the three elementary reactions constituting the acetic acid–oxaloacetic acid pericycle in the autocatalytic extension of the rTCA cycle. Each row shows the corresponding reactants, products, and optimized transition-state structures.

References:

1. Zhao Q, Garimella SS, Savoie BM. Thermally Accessible Prebiotic Pathways for Forming Ribonucleic Acid and Protein Precursors from Aqueous Hydrogen Cyanide. J Am Chem Soc. 2023 Mar 22;145(11):6135–43.

2. Zhao Q, Savoie BM. Algorithmic Explorations of Unimolecular and Bimolecular Reaction Spaces**. Angew Chem Int Ed. 2022 Nov 14;61(46):e202210693.

3. Kunkel JM, Yokota R, Taufer M, Shalf J, editors. High Performance Computing: ISC High Performance 2017 International Workshops, DRBSD, ExaComm, HCPM, HPC-IODC, IWOPH, IXPUG, P^3MA, VHPC, Visualization at Scale, WOPSSS, Frankfurt, Germany, June 18-22, 2017, Revised Selected Papers [Internet]. Cham: Springer International Publishing; 2017 [cited 2024 Sept 1]. (Lecture Notes in Computer Science; vol. 10524). Available from: http://link.springer.com/10.1007/978-3-319-67630-2

4. Ihlenfeldt W. PubChem. In: Engel T, Gasteiger J, editors. Applied Chemoinformatics [Internet]. 1st ed. Wiley; 2018 [cited 2025 May 3]. p. 245–58. Available from: https://onlinelibrary.wiley.com/doi/10.1002/9783527806539.ch6e

5. Zhao Q, Singla V, Hsu HH, Savoie B. Graphically-Defined Model Reactions are Extensible, Accurate, and Systematically Improvable [Internet]. 2025 [cited 2025 May 6]. Available from: https://chemrxiv.org/engage/chemrxiv/article-details/67b3e1bdfa469535b9c934b3

6. Bannwarth C, Ehlert S, Grimme S. GFN2-xTB—An Accurate and Broadly Parametrized Self-Consistent Tight-Binding Quantum Chemical Method with Multipole Electrostatics and Density-Dependent Dispersion Contributions. J Chem Theory Comput. 2019 Mar 12;15(3):1652–71.

7. Hsu HH, Jin T, Savoie B. Reaction Exploration Reveals Strong Kinetic Filtering in Li-Ion Battery Electrolyte Degradation [Internet]. 2025 [cited 2025 May 29]. Available from: https://chemrxiv.org/engage/chemrxiv/article-details/67f3de19fa469535b92071a2

8. Stubbs RT, Yadav M, Krishnamurthy R, Springsteen G. A plausible metal-free ancestral analogue of the Krebs cycle composed entirely of α-ketoacids. Nat Chem. 2020 Nov;12(11):1016–22.

9. Muchowska KB, Chevallot-Beroux E, Moran J. Recreating ancient metabolic pathways before enzymes. Bioorg Med Chem. 2019 June;27(12):2292–7.

10. Muchowska KB, Varma SJ, Moran J. Nonenzymatic Metabolic Reactions and Life's Origins. Chem Rev. 2020 Aug 12;120(15):7708–44.

11. Orgel LE. The Implausibility of Metabolic Cycles on the Prebiotic Earth. PLoS Biol. 2008 Jan 22;6(1):e18.
